# Heart rate variability parameters and fetal movement complement fetal behavioral states detection via magnetography to monitor neurovegetative development

**DOI:** 10.3389/fnhum.2015.00147

**Published:** 2015-04-07

**Authors:** Johanna Brändle, Hubert Preissl, Rossitza Draganova, Erick Ortiz, Karl O. Kagan, Harald Abele, Sara Y. Brucker, Isabelle Kiefer-Schmidt

**Affiliations:** ^1^University Women’s Hospital and Research Institute for Women’s Health, University of TuebingenTuebingen, Germany; ^2^fMEG Center, University of TuebingenTuebingen, Germany; ^3^Department of Obstetrics and Gynecology, University of TuebingenTuebingen, Germany

**Keywords:** fetal behavioral states, heart rate variability (HRV), fetal magnetocardiography (fMCG), fetal maturation, autonomic nervous system (ANS)

## Abstract

Fetal behavioral states are defined by fetal movement and heart rate variability (HRV). At 32 weeks of gestational age (GA) the distinction of four fetal behavioral states represented by combinations of quiet or active sleep or awakeness is possible. Prior to 32 weeks, only periods of fetal activity and quiesence can be distinguished. The increasing synchronization of fetal movement and HRV reflects the development of the autonomic nervous system (ANS) control. Fetal magnetocardiography (fMCG) detects fetal heart activity at high temporal resolution, enabling the calculation of HRV parameters. This study combined the criteria of fetal movement with the HRV analysis to complete the criteria for fetal state detection. HRV parameters were calculated including the standard deviation of the normal-to-normal R–R interval (SDNN), the mean square of successive differences of the R–R intervals (RMSSD, SDNN/RMSSD ratio, and permutation entropy (PE) to gain information about the developing influence of the ANS within each fetal state. In this study, 55 magnetocardiograms from healthy fetuses of 24–41 weeks’ GA were recorded for up to 45 min using a fetal biomagnetometer. Fetal states were classified based on HRV and movement detection. HRV parameters were calculated for each state. Before GA 32 weeks, 58.4% quiescence and 41.6% activity cycles were observed. Later, 24% quiet sleep state (1F), 65.4% active sleep state (2F), and 10.6% active awake state (4F) were observed. SDNN increased over gestation. Changes of HRV parameters between the fetal behavioral states, especially between 1F and 4F, were statistically significant. Increasing fetal activity was confirmed by a decrease in PE complexity measures. The fHRV parameters support the differentiation between states and indicate the development of autonomous nervous control of heart rate function.

## Introduction

Nijhuis et al. (1982) classified fetal behavior after 32 weeks of gestation into four states (quiet sleep 1F, active sleep 2F, quiet awake 3F, active awake 4F, see **Table 1**), based on fHRV, eye movement and body movement measured by CTG, and ultrasound (Prechtl, 1985; Drogtrop et al., 1990; Nijhuis et al., 1999). Prior to 32 weeks of GA, it is possible to distinguish activity and resting cycles (Pillai and James, 1990b). Fetal heart rate and movement is a common obstetrical marker of fetal well-being and health*.* Starting in the 1980s, fetal behavioral states were introduced as a concept based on fetal heart rate and movement classification and information was discovered about the development of the ANS during pregnancy.

**Table 1:** Criteria of the automatic state classification based on the original Nijhuis criteria.

State/fHRP	1F/fHRP1 quiet sleep	2F/fHRP2 active sleep	4F/fHRP4 active awake
Original criteria	• Quiescence which can be regularly interrupted by brief body movements (startles) • Stable heart rate, small oscillation • Isolated accelerations occur strictly related to movement	• Frequent gross body movement Heart rate with wider bandwidth than 1F • Frequent accelerations during movement	• Vigorous activity with many trunk rotations • Unstable heart rate • Large and long lasting accelerations fused into sustained tachycardia
**Criteria for automatic state detection**
Baseline	<160 bpm	<160 bpm	>160 bpm possible
Oscillation bandwith	<±7.5 bpm	±7.5–±15 bpm	>±15 bpm
Accelerations	No	>15 bpm/>15 s	>30 bpm/>30 s
Movement	No	Yes	Yes


Fetal magnetocardiography uses SQUID biomagnetometry to non-invasively record fetal heart function through the maternal abdomen. This method detects the fetal cardio electrophysiology with high temporal resolution (1 ms) superior to CTG and is less susceptible to artifacts than fECG (Peters et al., 2001). Thus, an exact detection of the fetal HRV is possible. Several research groups have confirmed the usefulness of fMCG as a new and safe technique for prenatal evaluation of fetal well-being and neurovegetative development by fHRV analysis (Van Leeuwen, 2004; Schneider et al., 2008). Most of the prior fMCG studies were conducted with small-array biomagnetometer systems, using visual classification of the data to identify fetal behavioral states. In 2008, a fetal magnetography system was installed at the MEG Center Tuebingen, dedicated for fetal monitoring. The system succeeds an earlier fetal system and provides improved signal acquisition for fetal assessment with enhanced detection of fetal signals (Lowery et al., 2006), making it possible to record fetal heart signals fMCG with high temporal resolution and fetal brain activity fMEG with high detection rates (Preissl et al., 2004). The characterization of normal fetal behavior is fundamental to neurodevelopmental research and clinical fetal evaluation. Fetal heart rate is influenced by the ANS, which matures during pregnancy. In addition several HRV parameters express the maturing influence of both ANS branches (sympathetic and parasympathetic). The SDNN measures the overall variability of the neurovegetative system. The RMSSD represents the short-term variability associated with vagal function. The SDNN/RMSSD ratio reflects sympathovagal balance (Schneider et al., 2008), and PE represents the complexity of heart beat intervals (Frank et al., 2006). Due to the high temporal resolution of the MCG, these parameters can be reliably estimated and could improve HRV analysis to enable the monitoring of the current fetal neurovegetative state, as shown in an earlier fMCG study (Schneider et al., 2008).

The focus of this study was the inclusion of fetal movement data according to the original Nijhuis criteria for fetal state classification. Additionally, HRV parameters (SDNN, RMSSD, ratio SDNN/RMSSD, PE) were simultaneously studied to gain information about the developing influence of the ANS within each fetal state. This was done by adapting the design of an earlier fMCG study (Schneider et al., 2008). An algorithm was used for an automatic fetal behavioral state detection – in order to provide a reproducible and objective approach, visual state detection was used to control and verify the results. Further interest should focus on the combination of the neurodevelopmental information obtained by both fMCG and fMEG, measuring fetal brain activity in utero, for the future, clinical applications of fetal magnetography.

## Materials and Methods

### Subjects

The study was performed with the fetal biomagnetometer installed at the fMEG Center in Tuebingen, Germany (Kiefer-Schmidt et al., 2013; Sonanini et al., 2014). The Ethics Committee of the Medical Faculty of the University of Tuebingen, Germany, approved the study. Written informed consent was obtained from all subjects. Fifty-five fetal magnetography recordings were obtained between 24 and 41 weeks of GA from women with uncomplicated, healthy singleton pregnancies. Subjects were divided into three GA groups: group 1 (GA 24+0 to 32+0 weeks), group 2 (GA 32+1 to 35+0 weeks), and group 3 (GA 35+1 to –41 weeks). Chromosomal abnormalities, fetal infections, and maternal diseases with negative effects on the unborn child were excluded. Only fetuses with estimated weights between the 10th and 90th percentiles for GA as determined by ultrasound were included in this study. Neonatal outcome was obtained after delivery to confirm a health child.

A CTG was routinely performed before every fMCG recording to confirm fetal heart rate and activity as normal for GA. Furthermore, the fetal position in relation to the sensor array was checked by ultrasound (Logiq 500 MD, GE Healthcare, Little Chalfont, Buckinghamshire, UK) prior to the recording. The study was performed in a magnetically shielded room (Vakuumschmelze, Hanau, Germany). The sensor array consisted of 156 SQUIDs (first order gradiometers) and 29 reference channels for noise detection (CTF MEGTM System, VSM Med. Tech, Coquitlam, BC, Canada). Subjects were seated comfortably in an upright position and asked to lean forward against the concave sensor array, modeled especially for the pregnant abdomen. Four coils fixed on elastic belts were positioned around the maternal abdomen to mark fetal head position with respect to the sensor array and to detect maternal movement during the measurement. The mothers were asked to relax during the recording and to move as little as possible. A choice of relaxing music was offered and transferred via air-conducting lines from a music player outside the room to a headphone. The duration of the recording depended on maternal comfort and was set to a maximum of 45 min. Subsequently, the ultrasound examination was repeated to check fetal position.

### Data Acquisition

**FIGURE 1:**
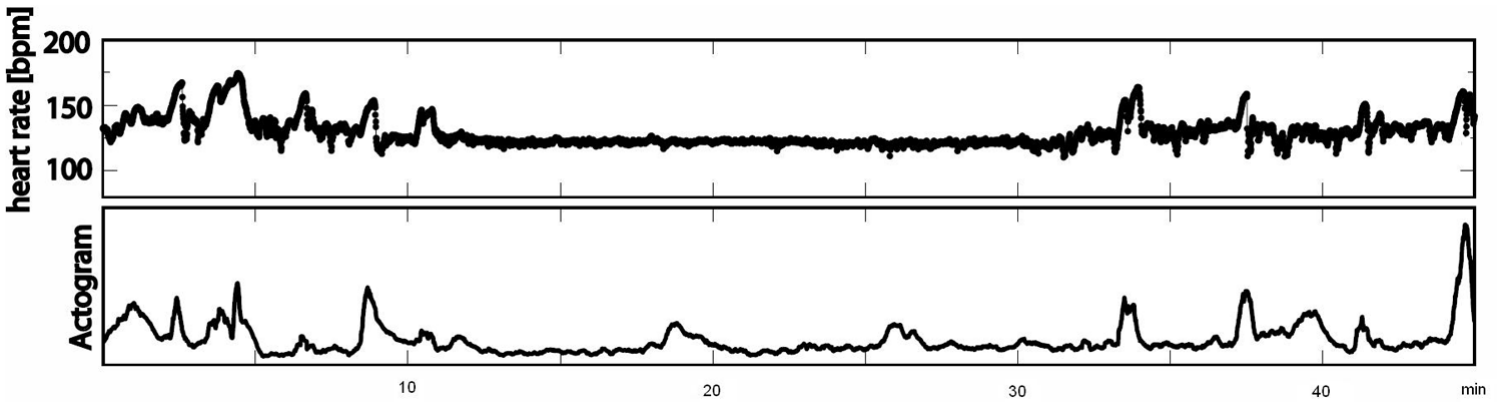
**Example of an actocardiogram in 38 weeks of GA measured over 45 min (first line: cardiogram in bpm; second line: actogram)**.

The recordings were performed at a sampling rate of 1220.7 Hz. Datasets with low signal-to-noise ratios for fetal heart signals and data with more than 3% artifacts or missed heartbeats were excluded from the analysis. All data were filtered with a bandpass of 1–80 Hz using the 8th order Butterworth filter with zero-phase distortion. Maternal heart signals were attenuated using a signal space projection technique and the fetal R-waves were identified using the Hilbert transform technique. The time between two R-waves was defined as a beat-to-beat interval and used to calculate fetal mHR. Classical parameters of fHRV representing the time domain (SDNN, RMSSD, SDNN/RMSSD ratio) and a non-linear fHRV measure (PE) were calculated for each state, 1F through 4F, and each gestational group in a moving window of 256 bpm. As a preprocessing step, a shifting window with a fixed size of 256 heartbeats was standardized in accordance with recommended standards (Malik, 1996; Grimm et al., 2003). The HRV parameters calculated were: SDNN – the standard deviation of normal-to-normal beats – representing the overall variability of sympathetic and vagal oscillations in the short data windows; RMSSD – the root mean square of successive differences, reflecting vagal control; the SDNN/RMSSD ratio – relating overall variability to its short-term variability shared in the time domain as a measure of sympathovagal balance (Schneider et al., 2008); and PE, representing the complexity of heart rate series (Frank et al., 2006). Fetal heart rate over time was plotted in bpm as a cardiogram in a CTG-like fashion. The fMCG signal measures fetal movement as changes in the orientation of fetal heart vectors with respect to the sensor array. This detection of fetal movement is orientated solely on the fetal heart vector and therefore only gross fetal movements such as trunk rotations are discernible. The resulting variation in signal amplitude was plotted as an actogram showing the fluctuation of the baseline over time. Any deviation >25% from baseline was considered to represent fetal movement. The cardiogram and the actogram were recorded simultaneously and plotted together as an actocardiogram, plotted in **Figure 1**. We developed an algorithm for automatic state classification based on the Nijhuis criteria (**Table 1**), taking into account the occurrence of fetal movement and the fHRP. All datasets were additionally classified by visual inspection of the actocardiograms by an observer with experience in the analysis of CTG and actocardiograms. A second observer with experience further independently analyzed the actocardiograms. If disagreement occurred, consensus was achieved by revision. Due to the low occurrence of the 3F state (Schneider et al., 2008), only the 1F, 2F, and 4F states were included in the present analysis. Prior to GA 32 weeks (group 1), only active and quiet states were distinguished, corresponding to the algorithm criteria of 1F for quiescence and 2F for activity (Pillai and James, 1990b).

### Statistics

Statistics were performed with SPSS 18.0 for Windows (IBM, Armonk, NY, USA). A one-way ANOVA was used for the statistical analysis of fetal behavioral states (independent variable) and parameters of HRV (dependent variable; **Figures 3 and 4**). To improve the design of**Figures 3 and 4** all data were plotted on a logarithmic axis. A value of p < 0.05 was considered statistically significant. *Post hoc* analysis of the difference between the individual states and three age groups employed the Mann–Whitney *U* test. After correction for multiple comparisons (Bonferroni), *p* < 0.0167 was considered significant for the *post hoc* analysis. The correlation of fHRV parameters with GA was analyzed by Spearman’s rank correlation.

## Results

**FIGURE 2:**
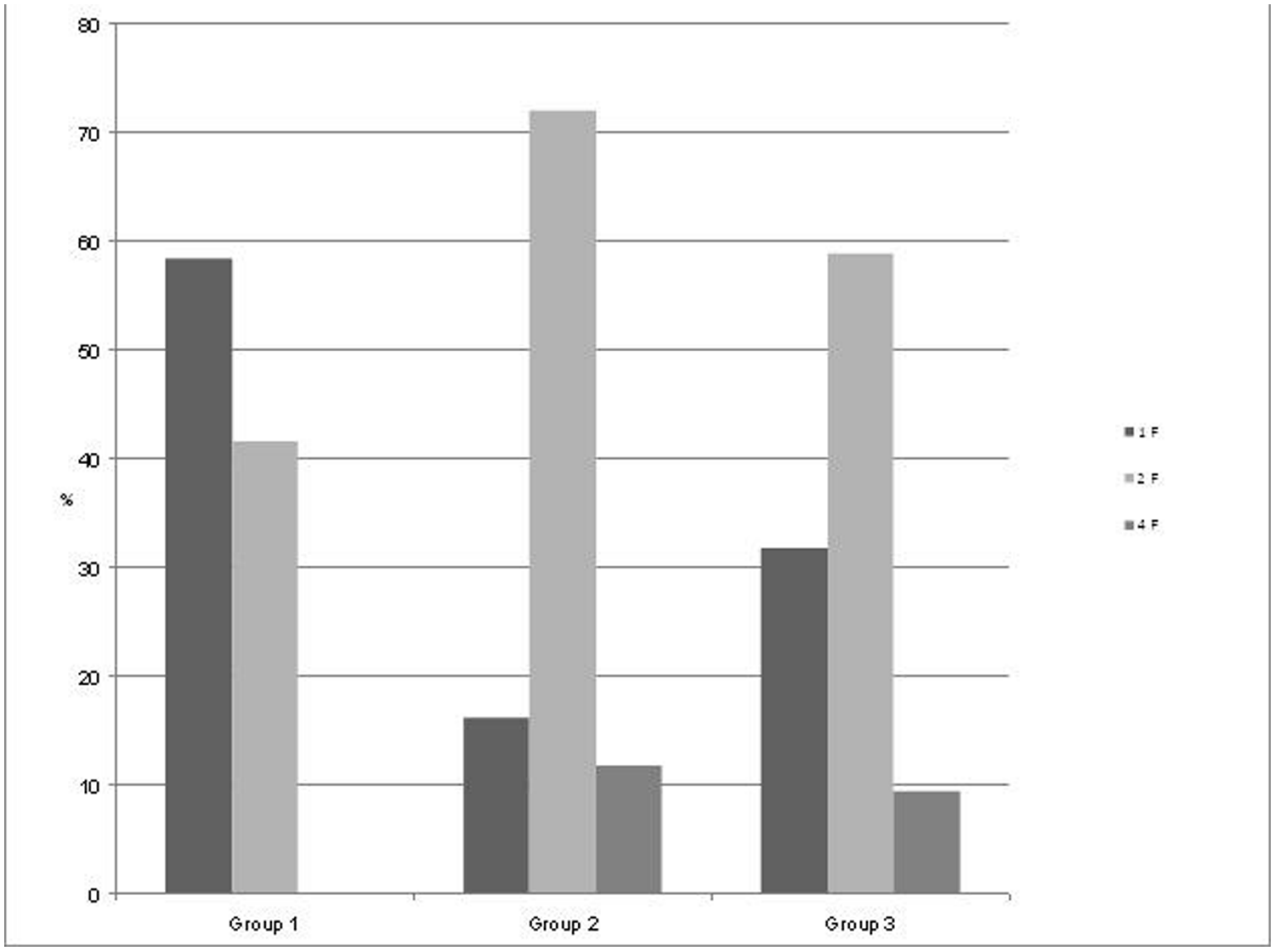
**Distribution of the fetal behavioral states in percent of total recording time per GA group.** Group 1: GA 24+0 to 32+0 weeks), Group 2: GA 32+1 to 35+0 weeks, and Group 3: GA 35+1 to 41 weeks.

Starting at 24 weeks of GA, we performed measurements in 55 pregnant women (mean age 33 years) with a mean recording time of 32.5 min (range 10–45 min) divided into three groups by GA (group 1, *n* = 18; group 2, *n* = 15; and group 3, *n* = 22) as seen in **Figure 2**. State detection was possible in all 55 datastets (automatic state classification: *n* = 49; visual classification: *n* = 6). In 89% of all cases automatic state classification was used successfully. Only in 11% (*n* = 6) the algorithm failed and visual inspection was needed for classification. In this visual classification the two observers disagreed in two cases an a consensus was achieved by revision. In group 1 (GA < 32 weeks), fetuses were in the resting state and in activity cycles during 58.4 and 41.6% of the recording time, respectively. Group 2 (GA 32–35 weeks), exhibited 1F, 2F, and 4F during 16.2, 72, and 11.8% of the recording time. In the late gestation group 3, the occurrence of 1F increased to 31.8%, 2F decreased to 58.8% and 4F remained almost unchanged at 9.4% (**Figure 2**).

### Parameters of fHRV and GA

**FIGURE 3:**
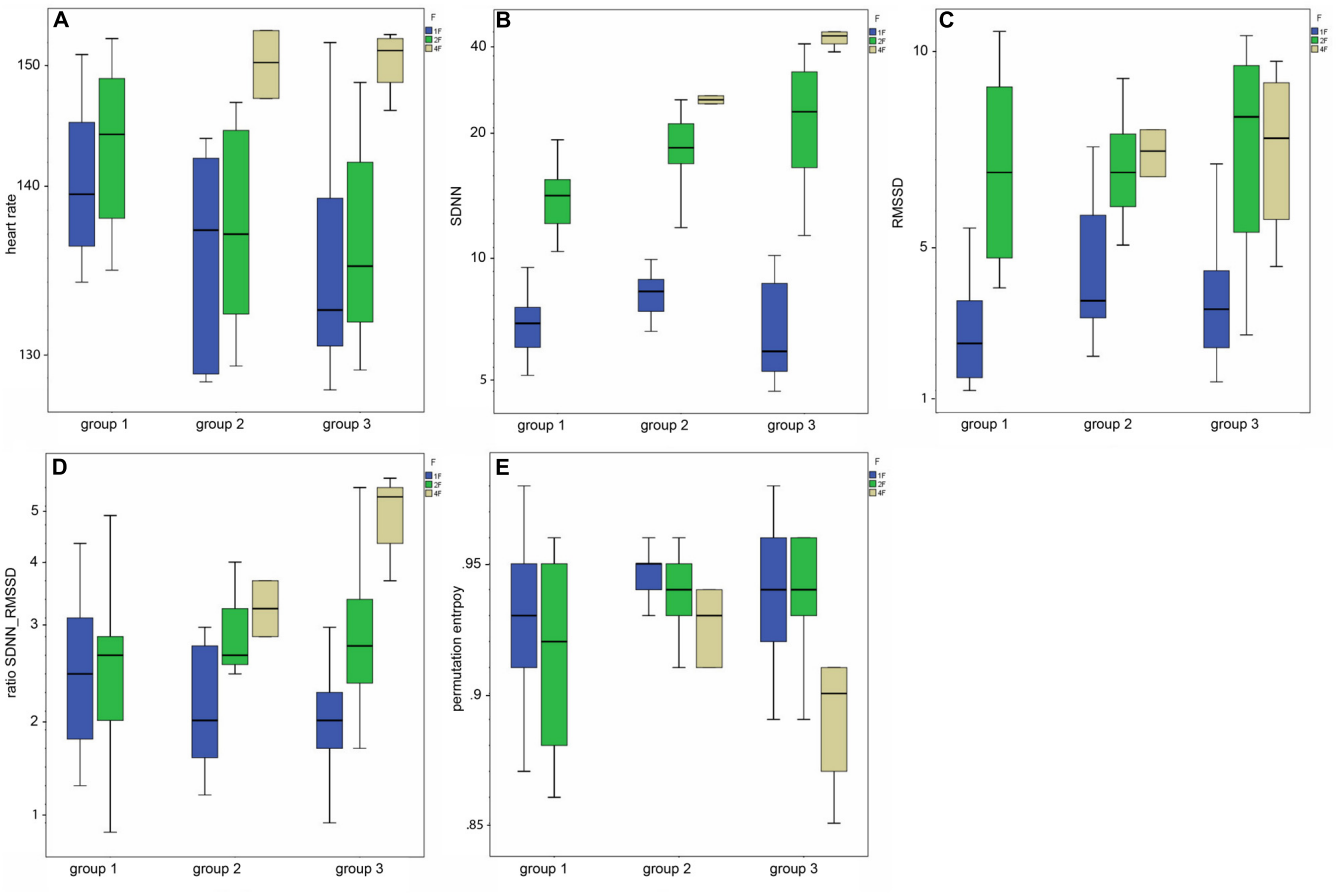
**(A–E)** Box-and-whiskers plots of the HRV parameters by fetal behavioral state (1F, 2F, 4F) and GA group (group 1–3).

Mean heart rate was stable between group 1 (144 bpm) and group 2 (145 bpm), but decreased to 141 bpm in group 3 (corr: -0.363, *p* < 0.001), as demonstrated in **Figure 3A**. This shift was significant (χ^2^ = 12.48; *p* < 0.005) between the GA groups in general and between group 1 and 3 in the *post hoc* analysis.

The SDNN showed an increasing trend with GA for state 2F and 4F, as seen in **Figure 3B**, but did not attain statistical significance between the age groups in general (χ^2^ = 5.43, *p* = 0.066). The increase was significant from group 1 to group 2, but not between the other age groups. There was no significant correlation between GA and SDNN in general (corr: 0.181; *p* = 0.081).

The RMSSD showed no clear decrease or increase across the GA groups, nor was the correlation between RMSSD and GA statistically significant (corr: 0.103; *p* = 0.323;**Figure 3C**).

The SDNN/RMSSD ratio (**Figure 3D**) showed a decreasing trend between group 1 and 2, group 2 and 3, and group 1 and 3 (corr: 0.026; *p* = 0.805).

PE did not show any significant changes between the GA groups (corr: 0.179; *p* = 0.059; **Figure 3E**). The decrease did not attain statistical significance as an overall main effect between all three groups (χ^sup2^ = 3.52, *p* = 0.172).

**Table 2**indicates the results of the *post hoc* analysis for each fHRV parameter between the age groups. **Figures 3A–E** shows the distribution of the HRV parameters by GA group. **Table 3** indicates the distribution of the fHRP parameter in the age groups.

**Table 2:** *Post hoc* analysis (*U =*Mann–Whitney test) of the HRV parameters and the age groups.

Group	mHR	SDNN	RMSSD	SDNN/RMSSD ratio	PE
1–2 1–3 2–3	0.032 (*U* = 199.0) **0.001(*U* = 350.0)** 0.400(*U* = 373.0)	**0.009 (*U* = 174.0)** 0.101 (*U* = 517.0) 0.941 (*U* = 425.0)	0.217 (*U* = 246.0) 0.306 (*U* = 573.0) 0.931 (*U* = 415.0)	0.041 (*U* = 0.707) 0.330 (*U* = 0.902) 0.990 (*U* = 1.5)	0.165 (*U* = 225.0) 0.080 (*U* = 506.5) 0.831 (*U* = 394.5)


Statistically significant *p-*values are in bold type.

**Table 3:** Distribution of the measured parameters of fHRP divided in groups regarding GA [Mean (STD)].

Group 1	Rest:	Active:
Mean HR	142.76 (5.10)	148.72 (7.36)
SDNN	7.48 (0.82)	18.77 (4.38)
RMSSD	3.23 (1.13)	13.15 (9.78)
Ratio SDNN/RMSSD	2.55 (0.82)	1.99 (1.26)
Perm. entropy	0.93 (0.03)	0.92 (0.03)

**Group 2**	**1F:**	**2F:**	**4F:**

Mean HR	136.34 (6.80)	141.02 (6.77)	156.92 (10.63)
SDNN	8.84 (1.22)	22.59 (3.93)	26.99 (10.39)
RMSSD	4.57 (1.44)	7.82 (3.57)	6.99 (2.04)
Ratio SDNN/RMSSD	2.10 (0.58)	3.33 (1.04)	4.06 (1.17)
Perm. Entropy	0.95 (0.02)	0.94 (0.02)	0.93 (0.03)

**Group 3**	**1F:**	**2F:**	**4F:**

Mean HR	134.21 (7.45)	137.83 (6.03)	150.82 (4.97)
SDNN	7.43 (1.57)	25.43 (4.87)	34.04 (10.83)
RMSSD	4.29 (1.14)	8.83 (3.46)	7.29 (4.55)
Ratio SDNN/RMSSD	1.84 (0.59)	3.28 (1.28)	5.41 (2.21)
PE	0.95 (0.02)	0.94 (0.02)	0.90 (0.03)

### Parameters of fHRV and Fetal Behavioral States

**FIGURE 4:**
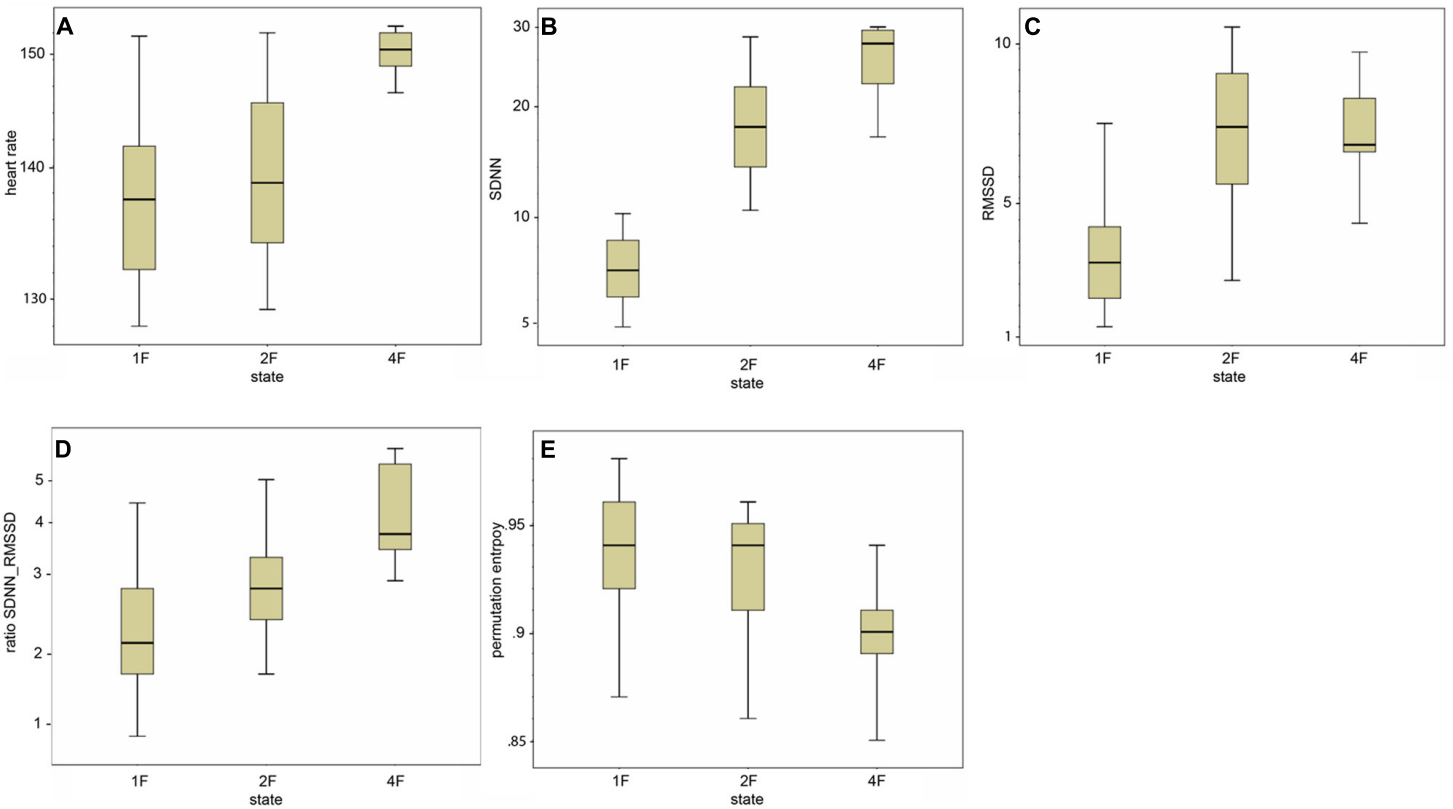
**(A–E)** Box-and-whiskers plots of the distribution of the individual HRV parameter per fetal behavioral state (1F, 2F, 4F).

Classical fHRV parameters were calculated for each recording in relation to the different fetal behavioral states. **Figures 4––E** shows the distribution of the HRV parameters by fetal behavioral state. As shown in **Table 4**, mHR increased from 1F to 2F, from 1F to 4F, and from 2F to 4F in each GA group. This main effect was statistically significant between the states for all fetuses in general (χ^2^ = 31.87, *p* < 0.001) and for the changes in behavioral state from 1F to 4F and from 2F to 4F. The SDNN increased significantly with the fetal behavioral state (χ^2^ = 95.42, *p* < 0.001). From 1F to 2F and from 1 F to 4F the increase attained statistical significance (**Figure 4B**). The RMSSD increased significantly with the behavioral state in general (χ^2^ = 54.19, *p* < 0.001) and from 1F to 2F and 1F to 4F, as seen in **Figure 4C**. The SDNN/RMSSD ratio increased significantly in general as a main effect (χ^2^ = 1.76, *p* < 0.001) and from 1F to 2F and 1F to 4F (**Figure 4D**). PE decreased significantly from 1F to 4F and 2F to 4F, but not from 1F to 2F (**Figure 4E**).

**Table 4:** *Post hoc* analysis (*U* = Mann–Whitney test) of the HRV parameter and the fetal behavioral states.

State changes	mHR	SDNN	RMSSD	SDNN/RMSSD ratio	PE
1F–2F	0.057 (*U* = 1444.0)	**0.000 (*U* = 1.00)**	**0.000 (*U* = 406.0)**	**0.000 (*U* = 0.002)**	0.721 (*U* = 1717.0)
1F–4F	**0.000 (*U* = 37.0)**	**0.000 (*U* = 13.0)**	**0.000 (*U* = 128.0)**	**0.000 (*U* = 0.101)**	**0.016 (*U* = 206.0)**
2F–4F	**0.000 (*U* = 67.0)**	0.030 (*U* = 314.0)	0.713 (*U* = 466.0)	0.042 (*U* = 0.674)	**0.009 (*U* = 273.5)**

*Statistically significant p-values are shown in bold type.*

## Discussion

### Fetal Behavioral States

Behavioral states in mature normal fetuses were primarily investigated by ultrasound relating to the original Nijhuis criteria, namely fHRPs, eye movement, and general body movement (Nijhuis et al., 1982). Between 36 and 42 weeks of GA, fetal behavioral states 1F, 2F, and 4F were reported as occurring 30.2, 57.5, and 9.5% of the time (Pillai and James, 1990a), respectively. This is in good agreement with our findings for the corresponding GA group 3 with 31.8% 1F, 58.8% 2F, and 9.4% 4F. Relating to the same gestational period, a fMCG study (Lange et al., 2009) visually classified fHRPs and found respective relative durations of 27.5, 42.5, and 20% for 1F, 2F, and 4F. The remaining 10% for 3F and the fact that the study used only fetal heart rate to classify fetal behavioral states without taking fetal movement into account might explain the differences in findings compared with our current study. Another fMCG study (Schneider et al., 2008) investigated fHRPs in the same GA groups as we did in our study and detected more 1F and 4F, but less 2F compared to our results in fetuses over 32 weeks of GA. Nevertheless, that study reported changes in fetal behavioral states with increasing GA from group 2 to group 3 that were similar to those observed in our present study, i.e., an increase in 1F (28–43.9% vs. 16.2–31.8% in our study) and decreases in 2F (50–42.1% vs. 72–58.8% in our study) and 4F (22.4–14% vs. 11.8–9.4% in our study). We aimed to describe fetal behavioral states more accurately by additionally taking into account fetal movement as one of the original Nijhuis criteria defining these states. The strong concordance between the behavioral state frequencies in mature fetuses beyond 36 weeks of GA and traditional studies of fetal behavioral state based on all Nijhuis criteria support our approach (Pillai and James, 1990a; Nijhuis, 1993). This indicates that this is a valid approach for fetal state detection. The advantage of an automatic state detection is to be objective and reproducible. As this algorithm was newly applied but not established yet, we double-checked the data visually and preferred in case of discrepancy the visual detected state. More data of a comparison between both methods is necessary to validate the automatic state detection.

During early gestation, only quiet vs. active states were distinguishable, representing the premature fetus. With progressing gestation, heart rate patterns became more defined, and matched fetal movement. The frequencies of fetal behavioral states developed as expected (Pillai and James, 1990a; Schneider et al., 2008). These results confirm that, as the ANS matures, the fetus develops the ability to synchronize HRV and body movement and to develop fetal behavioral states.

### Heart Rate Variability

Our further goal was to assess neurovegetative modulation by comparing established parameters of fetal HRV, namely SDNN, RMSSD, SDNN/RMSSD ratio, and PE, with the fetal behavioral states 1F, 2F, and 4F across three GA groups. The total values of the HRV parameters were in accordance with an MCG study based on visual classification of fHRPs to identify behavioral states (Schneider et al., 2008), although the individual values were spread out over a wide range and clustered more clearly within the three GA groups as demonstrated in . In our study, the SDNN showed an increasing trend with GA, indicating the increasing modulation of autonomous nervous control. Schneider et al. (2008) confirmed a slight increase based on higher values for 2F and 4F in fetuses over 32 weeks of GA. Moreover, as previously reported (Zhuravlev et al., 2002; Schneider et al., 2008), the SDNN/RMSSD ratio showed a decreasing trend with GA, indicating that the level of vegetative control increased toward term. However, both RMSSD and PE showed no clear changes with progressing gestation either in our data or in previous studies (Schneider et al., 2008). However, there were changes in parameters between the behavioral states. We observed increases in SDNN and RMSSD between the quiet sleep state (1F) to the two active states (2F and 4F; ). Whereas the SDNN reflects the overall increase in variability in vegetative function with fetal activity, the RMSSD represents the progressive vagal influence on short-term-variability. Maturation of the ANS more strongly emphasized the sympathetic branches, as indicated by the increase in SDNN/RMSSD ratio with fetal state. The changes in RMSSD from 2F to 4F were inverse to the changes in SDNN; a slight decrease was visible but not statistically significant. This finding was confirmed by prior studies showing the same shift in fetuses over 35 weeks of GA (Schneider et al., 2008; Lange et al., 2009). Moreover, earlier studies also reported an inverse change in SDNN/RMSSD ratio and PE, with the lowest ratio in the 1F state being observed for the linear parameters and the highest for PE. Notably, our data revealed differences in SDNN and RMSSD between the 1F and 2F states and in PE between the 2F and 4F states. All parameters showed significant shifts between 1F and 4F. These changes are in agreement with earlier reports of high linear fHRV parameters and low complexity measures in high fetal activity (Frank et al., 2006; Schneider et al., 2008).

The fHRV parameters may help to differentiate between fetal behavioral states and indicate the neurovegetative modulation within each state, thus offering greater insight into the vegetative development in utero. This confirms other studies pointing to the SDNN as a distinguishing parameter (Frank et al., 2006; Lange et al., 2009), including a more recent study indicating large state-dependent changes in SDNN (Wallwitz et al., 2012). mHR declined from the youngest to the oldest GA group, which reflects the known decrease in baseline heart rate toward term. MHR clearly increased with fetal activity as seen toward 4F, the behavioral state characterized by long lasting heart rate accelerations and possibly sustained tachycardia (Nijhuis et al., 1982). We conclude that developmental changes in HRV indicate how the autonomous nervous system matures the fetus’s ability to develop behavioral states. These findings were evident for fetal age itself from the clear findings between the states but not with GA in our study. The findings from our data are in agreement with earlier studies in this field (Schneider et al., 2008; Lange et al., 2009). In contrast, studies investigating parameters over the course of pregnancy (Van Leeuwen et al., 1999; Lange et al., 2005) revealed an increase in linear parameters and complexity measures with GA, but it remains unclear whether the underlying state development is a bias influencing this change in parameters. However, in our study there was no subdivision into age groups for the analysis of the parameter changes between the behavioral states nor did we perform a subanalysis of the behavioral states across age groups due to the limited number of cases. Schneider et al. (2008) showed changes for SDNN, RMSSD, and PE that were similar to those in our study in general, but they were able to further elucidate that the shifts in the parameters from 1F to 2F concerned all fetal age groups, whereas the changes from 2F to 4F or from 1F to 2F became evident only during the later stages of gestation, mostly after 35 weeks. According to the standard criteria for fetal behavioral states, the increasing maturation of the fetal ANS causes fetal heart rate and movement to become increasingly synchronized. This synchronization is a known marker of fetal well-being and is used in the clinical monitoring of pregnant women. It is well known that fetal HRV can be affected by a number of factors, including GA, maternal medication, or fetal pathology (Van Leeuwen et al., 1999; Lange et al., 2005, 2009; Lowery et al., 2008). Cases of high-risk pregnancy, e.g., IUGR or preeclampsia, showed lower levels of fetal movement, absence of fetal heart rate synchronization, and movement, or decelerations in fetal heart rate (Schneider et al., 2006, 2009). Therefore, the combination of fetal actocardiogram and HRV parameters could be helpful in indicating fetal distress with the aid of fetal magnetography. The use of fHRV parameters enhances the possibilities to monitor fetal autonomous nervous development making the combination of fMCG and fMEG expedient in future neurodevelopmental studies. This could serve as a starting point for the implementation of fetal magnetography as a multimodal tool for fetal assessment.

## Conflict of Interest Statement

The authors declare that the research was conducted in the absence of any commercial or financial relationships that could be construed as a potential conflict of interest.

## Abbreviations

ANS: autonomic nervous system
Bpm: beats per minute
CTG: cardiotocogram, cardiotocography, fECG, fetal Electrocardiography
fHRV: fetal heart rate variability
fHRP: fetal heart rate pattern
fMCG: fetal magnetocardiography
GA: gestational age
HRV: heart rate variability
IUGR: intrauterine growth restriction
mHR: mean heart rate
PE: permutation entropy
RMSSD: root mean square of successive differences of the R–R intervals
SDNN: standard deviation of the normal–to-normal R–R intervals
SQUID: standard deviation of the normal–to–normal R–R intervals
